# *Legionella* and the Prevention of Legionellosis

**DOI:** 10.3201/eid1406.080345

**Published:** 2008-06

**Authors:** Joseph E. McDade

**Affiliations:** * Rome, Georgia, USA

*Legionella* and the Prevention of Legionellosis is much less about the former and more about the latter. The book is essentially a risk-management manual for legionellosis, modeled on the World Health Organization’s framework for providing safe drinking water. Introductory chapters on the disease and the ecology of *Legionella* spp. and a concluding chapter on laboratory aspects of *Legionella* spp. serve as bookends for 7 chapters on risk management of legionellosis. The intervening chapters discuss known sources of risk for the disease: potable water; cooling towers and evaporative condensers; healthcare facilities; hotels and ships; and natural spas, hot tubs, and swimming pools. A chapter on disease surveillance and outbreak management and another on regulatory aspects complete the core.

Although this book is more a manual than a text, it has much to recommend and little to criticize. An international group of 58 experts contributed to the book, assuring consensus, completeness, and accuracy. Also, unlike many multiauthored texts, which typically suffer from duplication, frequent omissions, and widely varying writing styles, the book’s careful editing has averted these common pitfalls. However, an effort to ensure uniformity in some chapters has led to too much rigid conformity to style. An identical template is used for all risk-management chapters, and frequent use of bulleted lists is not particularly engaging and may prove insufficient for some readers.

The text is generously supplemented, perhaps overly so, with 33 tables, 14 figures, and 24 call-out boxes. However, the book’s front matter gives a listing of these illustrations for handy reference. Three appendixes are included: a sample water system checklist, a form for compiling relevant epidemiologic information about patients with Legionnaires’ disease, and an example of a national surveillance form. The list of references is impressive, and the glossary of terms will be valuable to many readers.

Notably, this disease-specific treatise arrives at a time when public health officials in some countries are moving toward an all-hazards approach to public health preparedness. Even within this context, this text will remain an authoritative reference for many years to come, and the generic algorithm for ensuring water safety has utility beyond the immediate scope of the book.

The brochure accompanying the book recommends it to environmental and public health officials, healthcare workers, workers in the travel industry, certain researchers, and perhaps some special interest groups. I concur with that general assessment, although the book will be used more frequently by some of those groups than by others.

